# Tristetraprolin specifically regulates the expression and alternative splicing of immune response genes in HeLa cells

**DOI:** 10.1186/s12865-019-0292-1

**Published:** 2019-05-02

**Authors:** Yafang Tu, Xiongfei Wu, Fengyun Yu, Jianzhong Dang, Juan Wang, Yaxun Wei, Zhitao Cai, Zhipeng Zhou, Wenliang Liao, Lian Li, Yi Zhang

**Affiliations:** 1 Nephrology Department, Renmin Hospital of Wuhan University, 238 Jiefang Road, Wuchang District, Wuhan, 430060 Hubei China; 2 Laboratory for Genome Regulation and Human Health, ABLife Inc., Optics Valley International Biomedical Park, East Lake High-Tech Development Zone, 388 Gaoxin 2nd Road, Wuhan, 430075 Hubei China; 3 Department of Geriatrics, Renmin Hospital of Wuhan University, 238 Jiefang Road, Wuchang District, Wuhan, 430060 Hubei China; 4 Center for Genome Analysis, ABLife Inc., Optics Valley International Biomedical Park, East Lake High-Tech Development Zone, 388 Gaoxin 2nd Road, Wuhan, 430075 Hubei China

**Keywords:** *ZFP36*, Overexpression, RNA-seq, Gene expression, Alternative splicing

## Abstract

**Background:**

Tristetraprolin (TTP) is an RNA binding protein that plays a critical role in regulating proinflammatory immune responses by destabilizing target mRNAs via binding to their AU-rich elements (AREs) in the 3′-UTRs of mRNAs. A recent CLIP-seq study revealed that TTP-binding sites are enriched in the intronic regions of RNA. TTP is also a nuclear protein that exhibits putative DNA-binding activity. These features suggested that TTP might regulate gene transcription and/or alternative splicing of pre-mRNAs in the absence of stimulation.

**Results:**

To elucidate the regulatory pattern of TTP, we cloned and overexpressed the human TTP-encoding gene, *ZFP36,* in HeLa cells in the absence of inflammatory stimuli. The transcriptomes of the control and *ZFP36-*overexpressing cells were sequenced and subjected to analysis and validation. Upon *ZFP36* overexpression, the expression of genes associated with innate immunity, including those in the type I interferon signaling pathway and viral response, were specifically upregulated, implying a transcriptional regulatory mechanism associated with the predicted DNA binding activity of TTP. TTP preferentially regulated the alternative splicing of genes involved in the positive regulation of the I-κB/NF-κB cascade and the TRIF-dependent toll-like receptor, MAPK, TNF, and T cell receptor signaling pathways.

**Conclusions:**

Our findings indicated that TTP may regulate the immune response via the regulation of alternative splicing and potentially transcription, which greatly expands the current understanding of the mechanisms of TTP-mediated gene regulation.

**Electronic supplementary material:**

The online version of this article (10.1186/s12865-019-0292-1) contains supplementary material, which is available to authorized users.

## Background

RNA binding proteins play key roles in coordinating RNA processing events (e.g., pre-mRNA splicing and polyadenylation, mRNA transport, and translation), creating vast opportunities for posttranscriptional gene regulation (PTGR) [1–3]. Moreover, RNA binding proteins are involved in regulating the establishment of a large array of physiological and pathological states [1, 4–9].

RNA binding proteins are well characterized for their ability to control multiple steps in the PTGR of the immune response [6, 8]. Among these, tristetraprolin (TTP) is one of the most well-studied RNA binding proteins [6, 8, 10]. In the early 1990s, TTP was identified as the prototype of a class of Cys-Cys-Cys-His (CCCH) zinc finger proteins, that was found to be proline-rich, widely distributed, and encoded by the immediate-early response gene, *ZFP36* [11, 12]. In addition, the transcription of *ZFP36* is rapidly accumulated in response to insulin [11] and growth factors [13]. In the late 1990s, experiments involving TTP-deficient mice indicated that it played a pathogenetic role and was linked to the cytokine, tumor necrosis factor alpha (TNFα) [14]. Moreover, the expression of both TTP and TNFα is induced upon stimulation with lipopolysaccharide (LPS), and TTP was shown to bind to the AU-rich element (ARE) of TNFα mRNA, promoting deadenylation, destabilization, and exerting a feedback inhibition [15, 16]. In subsequent studies, TTP was shown to target a number of other mRNAs associated with inflammation for degradation, most notably, cytokine and chemokine mRNA (e.g., IL-2, IL-3, IL-6, CCL2, CCL3, iNOS, COX2, and IL-10) [17–27]. Recently, CLIP technology has been applied to more accurately map the relationship between TTP-binding and its regulatory function in mouse macrophages following stimulation with LPS [28, 29].

TTP regulation of proinflammatory cytokine mRNA in macrophages is dependent on inflammatory conditions. Upon the induction of inflammation, TTP is phosphorylated by p38^MAPK^-activated protein kinase (MK2). The phosphorylation of TTP by MK2 results in the sequestration of TTP by 14–3-3 proteins and weakens the interaction of TTP with mRNA and CCR4-NOT, thereby stabilizing the target mRNA [30].

In addition to TTP, there are other RNA binding proteins involved in regulating the PTGR and RNA processing events associated with a proinflammatory immune response [31, 32]. For example, in contrast to TTP, HuR has been found to positively regulate the stability of several inflammatory cytokines, including IL-4, IL-13, IL-17, and TNF-α via binding to AU-rich elements [33]. In addition, RNA helicase DDX39B is a potent activator promoting the inclusion of *IL7R* exon 6 and consequently, a suppressor of the sIL7R protein isoform, which has been associated with an increased risk of multiple sclerosis [34]. The transcription of *CELF2* is induced during T cell signaling, which promotes widespread alternative splicing [35]. In addition, RC3H1 acts in concert with EDC4 and DDX6, whereas ZC3H12A associates with UPF1 to degrade mRNAs at spatiotemporally distinct phases of the inflammatory response [36].

Furthermore, the TTP-encoding gene, *ZFP36,* is also characterized as a cancer suppressor gene [37]. Most of the characterized suppressor functions of TTP have associated with its known target genes, including inflammatory cytokines (e.g., *IL8*, *IL6*, and *IL23*), as well as additional targets (e.g., vascular endothelial growth factor gene, *VEGF*) in melanoma cells [38], malignant glioma cells [39], head and neck squamous cell carcinoma [40], and colon cancer cells [41].

Most of what is currently known of TTP-mediated regulation of gene expression has been drawn from its ability to bind to the 3′-UTR as an ARE binding protein, which promotes target mRNA degradation. However, TTP is also located in the nucleus and mediates several DNA-binding activities. Stimulation with serum and other mitogens causes the rapid translocation of TTP into the cytosol, which is accompanied by rapid serine phosphorylation [13, 42, 43]. Furthermore, the global mapping of TTP binding sites in mouse macrophage transcripts has demonstrated its extensive binding to the intronic region of pre-mRNA [28]. However, it remains unclear as to whether nuclear-located TTP regulates any gene transcription or alternative splicing of pre-mRNAs in the absence of stimulation. Thus, the purpose of this study was to address these two related questions in HeLa cells rather than in immune responsive cells, which should eliminate the complications arising from the known function of TTP in regulating the immune response to inflammatory stimuli. To this end, we overexpressed the *ZFP36* gene in HeLa cells and analyzed the impact of TTP on the level of gene expression and alternative splicing in the absence of any inflammatory stimuli by sequencing and analyzing the transcriptomes of the overexpressing cells compared to the controls. Our results revealed that TTP could regulate the transcription and alternative splicing of a large number of genes involved in the innate immune and inflammatory response, which expands the current understanding of TTP-mediated immune regulation.

## Results

### RNA-seq data analysis

**Fig. 1:**
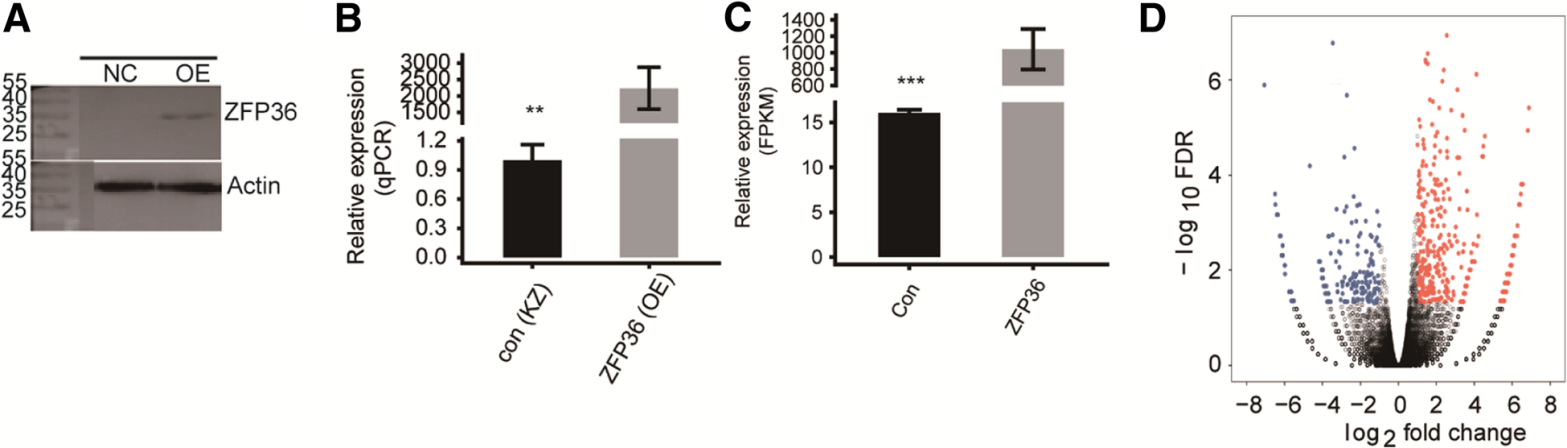
Differential gene expression in response to *ZFP36* overexpression. **a** HeLa cells were transiently transfected with either a *ZFP36*-overexpressing or control vector followed by determination of the level of FLAG-tagged TTP protein expression by western blot using an antibody against FLAG. **b** and **c** The FPKM values were calculated as described in the Methods. The level of mRNA expression was measured by qRT-PCR and RNA-seq. **d** Detection of the TTP*-* regulated genes on the volcano plots, up-regulated genes (FC ≥ 1.5; FDR < 0.05) are labeled red, whereas down-regulated (FC ≤ 2/3; FDR < 0.05) genes are labeled blue

To explore the molecular mechanism of TTP-mediated transcriptional regulation, RNA-seq experiments were performed. As shown in Fig. 1a and b, the efficacy of *ZFP36* overexpression was assessed by RT-PCR and western blots, approximately 10 times. Four cDNA libraries were constructed and sequenced using the Illumina Xten platform for 2 × 150 paired-end reads per sample.

After removing the adaptor sequences and low quality sequencing reads, we obtained a total of 98 ± 14.8 million high quality reads from each sample (Table 1). When these reads were mapped onto the human GRCH38 genome using Tophat2, 89.22–92.68% were aligned and about 95.46–96.8% were uniquely aligned (Table 1). To compare the gene expression patterns between individuals, we reassessed the gene and transcript quantifications with Cufflinks [44]. The expression values in units of fragments per kilo base of exon model per million fragments mapped (FPKM) was calculated. RNA-seq yielded robust expression results for 28,837 genes (see Additional file 1: Table S1). Effective overexpression of *ZFP36* was further confirmed in a parallel RNA-seq analysis (Fig. 1c). FPKM values for all 28,837 genes were used to calculate a correlation matrix based on the Pearson’s correlation coefficient.

**Table 1:** Summary of RNA-seq reads used in analysis

Sample	ZFP36_1st	ZFP36_2nd	Ctrl_1st	Ctrl_2nd	
Raw reads	97,277,834	122,099,646	85,307,388	103,111,480	101,949,087 + 15,342,348.1003348^a^
Clean reads	94,730,320	117,723,315	81,786,909	98,488,282	98,182,206.5 + 14,862,367.5115245
paired-end reads	92,564,654	114,105,262	78,689,244	94,366,960	94,931,530 + 14,575,832.6760188
Total mapped	84,903,807 (91.72%^b^)	105,756,131 (92.68%)	70,202,863 (89.22%)	85,742,261 (90.86%)	86,651,265.5 + 14,599,380.4773278
Total Uniquely mapped	81,485,850 (95.97%^c^)	101,851,278 (96.31%)	67,016,487 (95.46%)	82,999,189 (96.8%)	83,338,201 + 14,290,772.0956458
Splice reads	33,118,198 (40.64%^d^)	42,059,737 (41.3%)	28,029,484 (41.82%)	34,446,000 (41.5%)	

^a^The mean and standard deviation across the 4 samples

^b^the percentage of paired-end reads that were mapped to the genome

^c^the percentage of unique reads mapping out of the total mapped reads

^d^the percentage of uniquely mapped reads that were mapped to splice site

### *ZFP36* overexpression in HeLa cells preferentially upregulated the expression of a large number of immune response genes

**Fig. 2:**
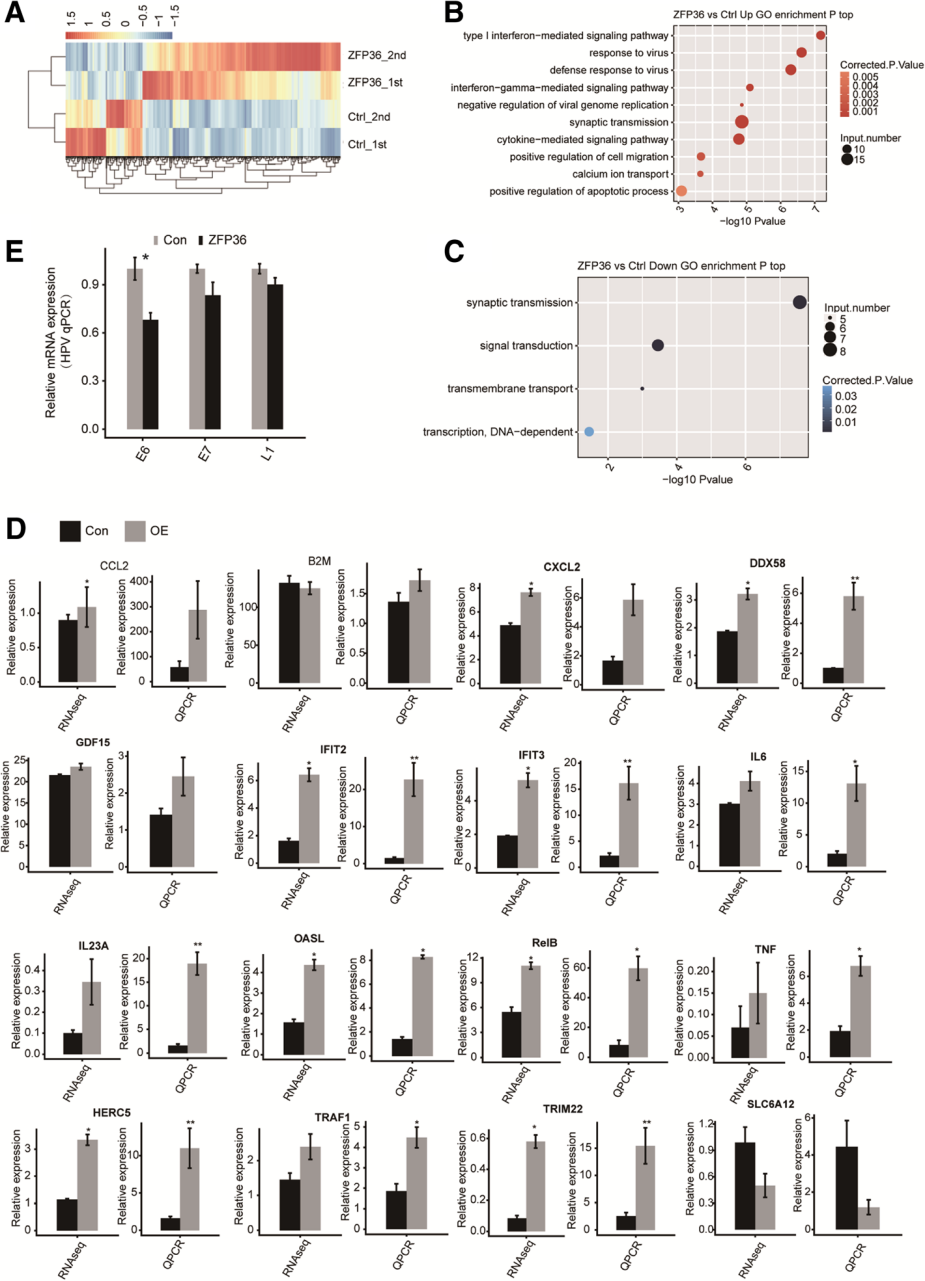
Analysis of the expression and the functional clustering of genes regulated by TTP. **a** Hierarchical clustering of the expression of 827 DEGs regulated by *ZFP36* overexpression in HeLa cells expressing either the control or the *ZFP36* plasmid. Expression values (FPKM) are log_2_-transformed and median-centered by each gene. **b** and **c** The top 10 representative GO biological process terms of the TTP-regulated genes. **d** Validation of DEGs by RT-qPCR. **e** The level of HPV gene expression was measured by qRT-PCR

Based on the high quality RNA-seq data obtained from the *ZFP36*-OE cells and the control, we used criteria of an absolute fold change ≥1.5 or ≤ 2/3, FDR < 0.05 with the edge R package [45] to identify genes that were potentially regulated by TTP at the transcriptional level. We identified 596 and 231 genes that were upregulated and downregulated, respectively. The details of the differentially expressed genes (DEGs) can be found in Additional file 2. A volcano plot was constructed to display the DEGs associated with *ZFP36* overexpression (Fig. 1d). A heat map analysis of the DEG expression patterns in the RNA-seq samples displayed high consistency with the TTP-mediated transcription in both data sets (Fig. 2a). These results indicated that TTP extensively regulated gene expression.

Based on the cut-off criteria, the upregulated DEGs were enriched in 66 GO terms, and the downregulated DEGs in 4 GO terms (see Additional files 3 and 4 for details). Strikingly, in the biological process terms of the GO analysis, the upregulated genes in the *ZFP36*-OE cells were primarily associated with the innate immune response, including the “type I interferon-mediated signaling pathway,” “response to virus,” “defense response to virus,” and “interferon-gamma-mediated signaling pathway.” The TTP up-regulated genes included, *IL23A, OASL, TRIM22, IFIT2, HERC5, IFIT3, IFI27, TNF, CSF2, IL6, CCL2, RELB, CXCL2, TRAF1,* and *DDX58*. TTP overexpression also resulted in the upregulation of genes enriched in the “cytokine-mediated signaling pathway” and “positive regulation of the apoptotic process” (Fig. 2b; for details see Additional file 4). In contrast, the downregulated genes were primarily enriched regarding “synaptic transmission.” The enrichment of genes involved in “signal transduction” was also observed (Fig. 2c).

When the corrected *p*-value for the Kyoto Encyclopedia of Genes and Genomes (KEGG) pathways were set at < 0.05; no pathways were enriched (Additional file 1: Figure S1; for details see Additional files 5 and 6). Surprisingly, when the adjusted *p*-value for the KEGG pathways were set at < 0.05, some genes were dysregulated in the TTP overexpressing samples annotated with KEGG categories and were mainly involved in the NF- κB signaling pathway (e.g., *RELB, DDX58, CXCL2, IL1R1,* and *TRAF1*) or chemokine signaling pathways (for details see Additional file 6).

We selected DEGs or non-DEGs involved in the immune response for further reverse transcription and quantitative real-time (qPCR) validation analysis. The qPCR results were highly consistent with the sequencing data (Fig. 2d and Table 2).

**Table 2:** primers used in q-PCR experiments

Overexpression				
ZFP36-F		CTGTCACCCTCTGCCTTCTC	ZFP36-R	TCCCAGGGACTGTACAGAGG
Differentially expressed genes				
IL23A-F		TCGGTGAACAACTGAGGG	IL23A-R	TCAAATCTGGCTGGCTCT
OASL-F		AGGCTGATATGGAAAACC	OASL-R	CTGTAGGCAGGCACAATG
TRIM22-F		GGAACCTCCGACCTAATC	TRIM22-R	ACAAACCCAGCAAATGAC
IFIT2-F		ATACATACCAAACAATGCCTAC	IRIT2-R	GAGCCACAGCGTGTCCTA
HERC5-F		GTGGAGAAATTGGGTATG	HERC5-R	TTAGGCTTGACAGGAAAC
IFIT3-F		ACACCAAACAATGGCTAC	IRIT3-R	AGGATTCAGTCCCTTCTC
TNF-F		CTCACATACTGACCCACG	TNR-R	AAGAGGCTGAGGAACAAG
IL6-F		CAGACAGCCACTCACCTC	IL6-R	CTCTTTGCTGCTTTCACA
CCL2-F		GAAAACTGAGGCACCAAG	CCL2-R	GGAGCTAGAGGAGGAACG
RelB-F		CCACGCCTGGTGTCTCGC	RelB-R	CGCTGCTTGGGCTGCTCC
CXCL2-F		CTGCGCTGCCAGTGCTTG	CXCL2-R	GCTTTCTGCCCATTCTTG
TRAF1-F		GGACCGTCAGCCTCTTCT	TRAR1-R	GCGCATCATACTCCCCTC
DDX58-F		CAGTGTATGAACAGCAGA	DDX58-R	GATGGATAGTGATGGAAT
B2M-F		GGTTTCATCCATCCGACATT	B2M-R	ACGGCAGGCATACTCATCTT
GDF15-F		CTTGTTAGCCAAAGACTGCC	GDF15-R	AACCTTGAGCCCATTCCACA
SLC6A12-F		GTGACACTCCCTTGGCTGGTG	SLC6A12-R	GCCTTCTTCATCCCCTACTT
HPV18-E6-F		ATAAGGTGCCTGCGGTGCC	HPV18-E6-R	TGCGTCGTTGGAGTCGTTC
HPV18-E7-F		GAGCACGACAGGAACGACT	HPV18-E7-R	GGGCTGGTAAATGTTGATGAT
HPV18-L1-F		CAGGTGGTGGCAATAAGCAGGA	HPV18-L1-F	TGGCGGCATGGGAACTTTCAG
GAPDH-F		GGTCGGAGTCAACGGATTTG	GAPDH-R	GGAAGATGGTGATGGGATTTC
Alternative splicing events				
TRIM38	AS1-F	TCGCCTCTGCTTATTCTA	AS1/AS2-R	CTTGATGACTCCTTAACATT
	AS2-F	CTTTGAGCAGGAGTTGGGCT		
IRF3	AS1/AS2-F	GAAATCCTCCTGCTGTGCATC	AS1-R	GACGCTGGAGGCCGGACCAT
			AS2-R	TCCATCGTAGGCCGGACCAT
TLR4	AS1/AS2-F	GGTCAGACGGTGATAGCG	AS1-R	TAGGAACCACCTCCACGCAG
			AS2-R	TGTGGTTTAGGGCCAAGT
LTBR	AS1/AS2-F	ACAAGCAAACGGAAGACC	AS1-R	CTGTGAGCCTCTTTGAGCTC
			AS2-R	GAGCTGCCTCCACCAGAC
NOD1	AS1 -F	GTTGTATAAACTTCAAGAGA	AS1/AS2-R	AAGGTGCTAAGCGAAGAG
	AS2-F	ATCCAGGATTTTGGTGAC		
CASP8	AS1/AS2-F	CCTGCTGGATATTTTCAT	AS1-R	AACTCCTCCCCTTTGCTG
			AS2-R	CTTCAAGGCTGCTGCTTC
MEF2A	AS1/AS2-F	GTAGCCCTTGGACTAGAAG	AS1-R	CCCAACCATTCTGTCCTATG
			AS2-R	TTCTTAAAGTCTGTCGGTTC
MAP2K7	AS1/AS2-F	CGGCGGCTCGACGGGGTC	AS1 -R	GGGCAGGAGCAGGGCTTAGAG
			AS2-R	AGCTGCAGGGTGGGCCTGGG
ATF2	AS1/AS2-F	CTGAAGCTTTCTTGAATTCATTCTC	AS1-R	ATGTGGCCAGATCAGACCCC
			AS2-R	GATCATTTGGCTGTCCATAAAC
FLNA	AS1/AS2-F	CTACTCATTTTGAGGCGCGAGAAGC	AS1-R	GACCAGAAGGACAACTTCAAGCCCG
			AS2-R	GGAACAGCAGCGCAACCTCT
KANK1	AS1/AS2-F	TTCCATCCTCTTCAGTTG	AS1-R	CTTCGTGTTTAAAGCCCTC
			AS2-R	GGCATTCAACCTCTCAACAA
SLC44A2	AS1-F	CAGTGCCTCCCTCCAG	AS1/AS2-R	TTGTAAATGGGTCCTTTG
	AS2-F	CGGCCATGGAGGACGAGC		
CARHSP1	AS1-F	ACAGCAAGTTAAAAAACAGTAGC	AS1/AS2-R	GGAAGCCTCCGGCCCTAT
	AS2-F	CATGGCTGACGTGTCTCCCG		
CREM	AS1/AS2-F	GGTCGGGCTCGCCGTCTC	AS1 -R	CACCACTTACCCGCCGCAAA
			AS2-R	AAGTTGGCATGTCACCAG
TEAD2	AS1/AS2-F	CTGGAGGATGGTGAAGTTTTCC	AS1-R	TTCCTCCAAGACGGAACGGG
			AS2-R	GGCAAGCAGGTGGTGGAG
MYCBP2	AS1/AS2-F	CTGAATGGTGAAATCCAC	AS1 -R	GAAGGAAGAGATGGAAGTGG
			AS2-R	TAAACGGGACAAGCACAAAG

Since HeLa is a well-characterized HPV+ cervical cancer cell line [46, 47], it could be possible that the activated expression of the immune response genes by *ZFP36* overexpression is due to an attempt to reactivate the expression of the HPV genome. To address this possibility, we detected the level of the HPV gene expression (E6, E7, and L1) by qRT-PCR analysis in *ZFP36*-overexpressed and control cells. None of the HPV genes displayed an increased expression upon *ZFP36* overexpression (Fig. 2e). The specific PCR primer pairs that were designed are presented in Table 2 [48]. Moreover, we also mapped the RNA-seq reads in this study onto the HPV genome, which demonstrated that extremely few reads originated from HPV and supported the lack of HPV activation. These results confirmed the lack of HPV activation upon *ZFP36* overexpression in HeLa cells.

### TTP positively regulates gene expression in HeLa cells without associating with the presence of an AU-rich element

**Fig. 3:**
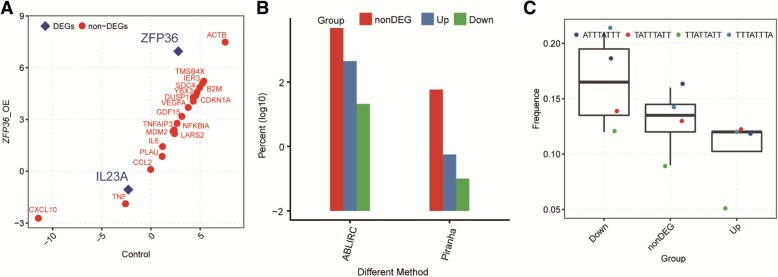
Analysis of the 3′-UTRs of genes upregulated by TTP. **a** Level of reported TTP target gene expression in HeLa cells. **b** Analysis the 3′-UTRs peak of the genes. **c** Analysis the 3′-UTR motifs of TTP-regulated or not regulated genes

TTP negatively regulates immune response genes by binding to the ARE elements located at the 3′-UTR of the mRNAs of these target genes, which leads to target mRNA degradation [10]. We hypothesized that the upregulated gene expression in HeLa cells should not reflect its binding to AU-rich elements in the 3′-UTRs of immune response genes. Consistent with this hypothesis, we found that apart from IL-23A, the level of all reported TTP target gene expression involving the binding of the mRNA AU-rich elements was not substantially altered (Fig. 3a). To correlate TTP-regulated gene expression with its 3′-UTR binding activity, we analyzed the previously published iCLIP data [28] and obtained mouse macrophage mRNAs containing TTP binding peaks at the 3′-UTR regions. We then calculated the percentage of TTP-regulated genes in HeLa cells whose mouse homologues contain a TTP binding peak in the 3′-UTR of mRNA in mouse macrophages. The statistical analysis showed that TTP-mediated down-regulated genes contained more 3′-UTR binding sites in mice, consistent with the view that TTP may downregulate gene expression in HeLa cells via its canonical 3′-UTR binding activity (Fig. 3b). To further test whether the TTP-regulated genes in HeLa cells are related to the AU-rich elements in the 3′-UTRs, we performed a motif search analysis, which showed that the frequency of AU-rich elements were substantially higher in the TTP-mediated downregulated genes (Fig. 3c). Taken together, these results confirmed that TTP may downregulate the expression of some genes in HeLa cells via its known 3′-UTR binding activity. Moreover, these findings are consistent with our hypothesis that TTP might upregulate gene expression at the transcriptional level, which may be distinct from the TTP-mRNA binding activity.

### TTP regulates the alternative splicing of a large number of immune-response genes

One key objective of this study was to gain an insight into the role of TTP in the regulation of alternative splicing. To use transcriptome data to explore the TTP-dependent AS events in HeLa cells, we assessed the data quality using an analysis of alternative splicing. Among the 83.3 M ± 14.2 M uniquely mapped reads from *ZFP36*-OE and control HeLa cells, 40.64–41.82% were junction reads (Table 1). When compared to the reference genome annotation, we found 68.66% annotated exons (252,220 out of 367,321 annotated exons), 166,260 annotated splice junctions, and 211,482 novel splice junctions by using the Tophat2 pipeline.

To investigate the global changes in the AS profiles in response to ZPF36 overexpression, AS events were analyzed from the RNA-seq dataset using ABLas software [49]. We detected 21,681 known alternative splicing events (ASEs) in the model gene that we termed the reference genome, and 49,136 novel ASEs, excluding intron retention events (Additional file 1: Table S2).

**Fig. 4:**
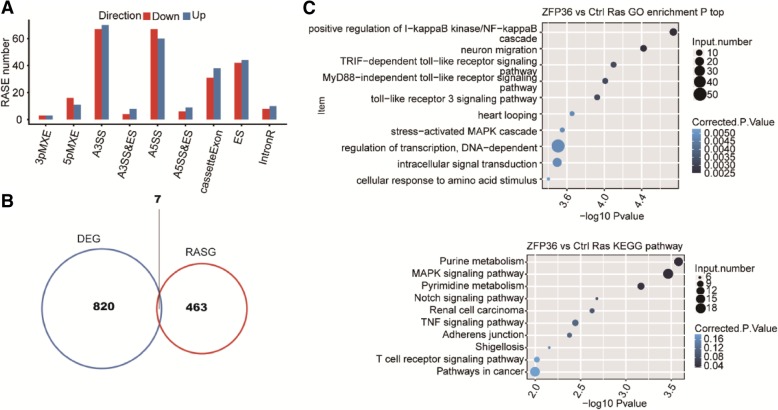
Identification and functional analysis of TTP-regulated alternative splicing events. **a** Classification of different AS types regulated by the TTP protein. **b** Overlap analysis between TTP-regulated genes (DEG) and alternative splicing genes (RASG). **c** The top 10 GO biological process analysis and KEGG functional pathway of alternative splicing gene

We applied a stringent cut-off *p*-value of ≤0.05, changed T-value ≥0.2 (See Methods) to identify regulated alternative splicing events (RASEs) with a high confidence, which resulted in 497 RASEs (the complete RASEs can be found in Additional files 7 and 8). TTP-regulated AS events included the Cassette exon (69)/exon skipping (86 ES), alternative 5′ splice site (127 A5SS), and alternative 3′ splice site (137 A5SS) (Fig. 4a). These data suggested that TTP globally regulated alternative splicing events in HeLa cells. To analyze whether the increase in AS events could be attributed to altered transcription [50], we overlapped the genes whose level of expression and alternative splicing were both regulated by TTP, and identified seven such genes: *GARNL3, PVRL4, ABCA5, TTC21A, HES4, LRRC37A11P,* and *SLX1B-SULT1A4* (Fig. 4b). This finding indicated that transcriptional regulation and alternative splicing might be partially coupled.

It was further revealed that the genes regulated by TTP-mediated alternative splicing were highly enriched for “positive regulation of I-kappaB kinase/NF-kappaB,” “TRIF-dependent toll-like receptor signaling pathway,” “MyD88-independent toll-like receptor signaling pathway,” “toll-like receptor 3 signaling pathway,” and “regulation of transcription, DNA-dependent” (GO biological process terms, Fig. 4c). Enriched KEGG pathways (*p* < 0.05) included those involved in “Renal cell carcinoma,” “Notch signaling pathway,” and “TNF signaling pathway” (Fig. 4c).

**Fig. 5:**
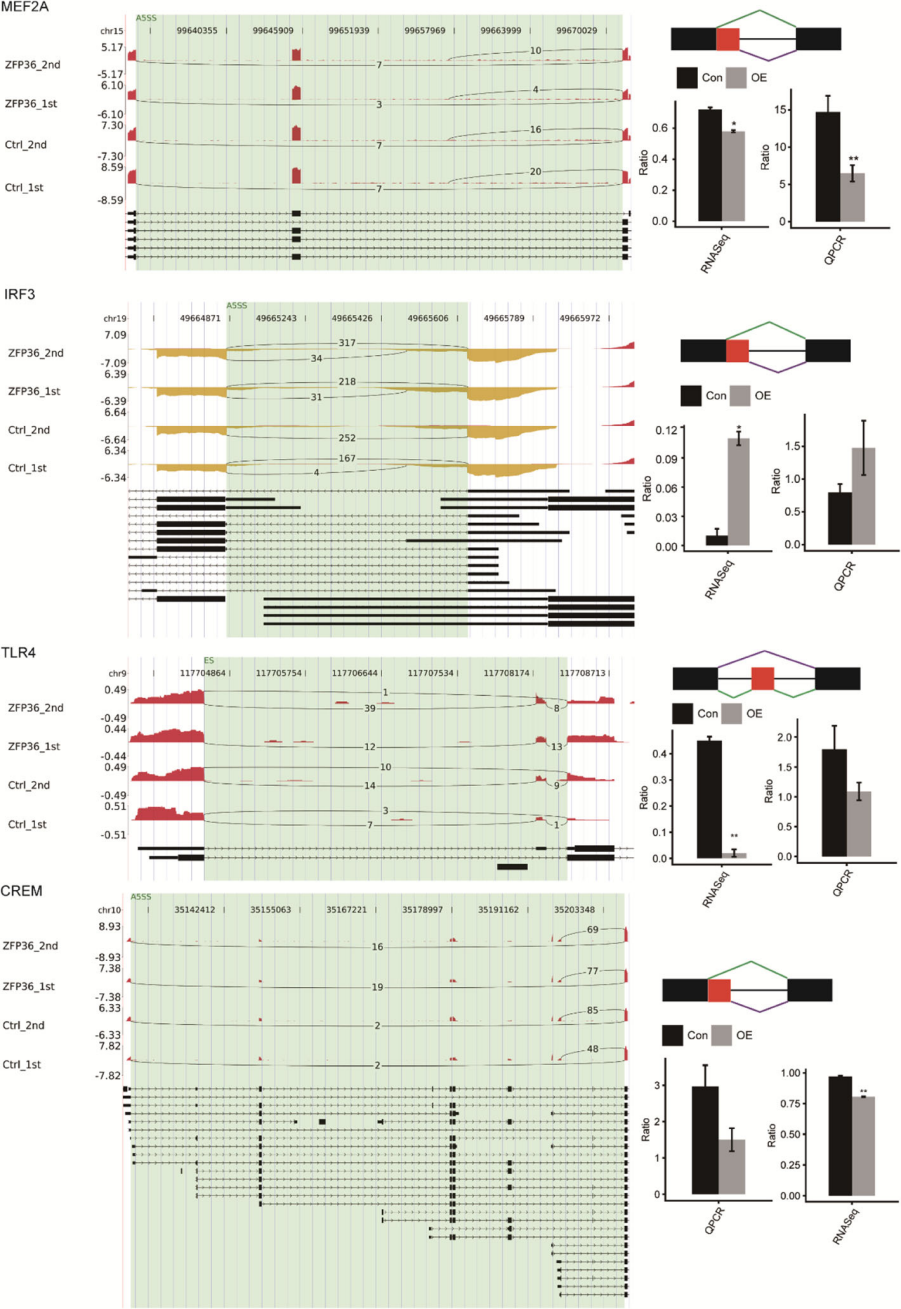
Validation of TTP-mediated AS events. (Left panel) IGV-sashimi plots showing the alternative splicing changes that occurred in the control or *ZFP36*-OE HeLa cells. The results for MEF2A, IRF3, TRL4, and CREM are presented. For each gene, the left panel shows the transcript isoforms of each gene (bottom) and the density map of all RNA-seq read distribution around the gene regions involved in the alternative splicing events (top) from the four samples. Alternative splicing isoforms of each AS event are depicted. The number of reads supporting each isoform are shown (top). In the right panel of each gene, the schematic diagram (top) depicts the structure of each alternative splicing event, AS1 (shown in purple) and AS2 (shown in green); exon sequences are denoted by boxes and intron sequences by the horizontal line. RNA-seq quantification and RT-PCR validation of alternative splicing regulation is shown at the bottom. The altered ratio of AS events in the RNA-seq were calculated using the formula: AS1 junction reads/AS1junction reads + AS2 junction reads. The altered ratio of AS events assessed by q-PCR were calculated using the formula: AS1 transcript level/ AS2 transcript level. The transcripts for the genes are presented below

To validate the alternative splicing events identified by the RNA-Seq data, 16 potential alternative splicing events were analyzed by q-PCR. PCR primer pairs (Table 2) were designed to amplify the long and short splicing isoforms in the same reaction. Out of 16 tested events, 13 alternative splicing events validated by q-PCR were in agreement with the RNA-Seq results. The 13 validated splicing events were located in the following genes (*TRIM38, IRF3, TLR4, LTBR, CASP8, MEF2A, MAP2K7, ATF2, SLC44A2, CARHSP1, CREM, TEAD2,* and *MYCBP2*) (Fig. 5 and Additional file 1: Figure S2).

## Discussion

To our best knowledge, this study is the first to profile the entire transcriptome in a nonimmune cell line (HeLa cells) with the overexpression of *ZFP36*, which allows for the decoding of TTP-mediated regulation of gene expression and alternative splicing in a system unrelated to inflammatory stimulation. Interestingly, upon *ZFP36* overexpression, the expression of genes associated with innate immunity, including those in the type I interferon signaling pathway and viral response, were specifically upregulated. In the absence of inflammatory stimuli, the upregulated expression of immune response genes were contradicted to the mRNA destabilization function of TTP via binding to the AU-rich element of the mRNA. Therefore, it is highly likely that TTP was able to promote the expression of immune response genes via a transcriptional regulatory mechanism associated with the predicted DNA binding activity. Furthermore, TTP preferentially regulated the alternative splicing of genes enriched in the positive regulation of the I-κB/NF-κB cascade, TRIF-dependent toll-like receptor signaling pathway, as well as the MAPK, TNF, and T cell receptor signaling pathways. Furthermore, our study indicated that TTP modulated the immune response via regulation of transcription and alternative mRNA splicing, thereby expanding our current understanding of the central role of TTP in regulating the immune response.

TTP is well-known for its capacity to regulate the mRNA stability of pro-inflammatory genes. This regulatory mechanism has been heavily used in the literature to explain its multiple roles in various physiological and pathological immune states [20–27]. However, a recent genome-wide study revealed that a large fraction of TTP binding events are not sufficient to drive mRNA destabilization [28]. Moreover, TTP binding of the intronic regions of pre-mRNAs is significant [28, 29], albeit with little reported function. Moreover, TTP-mediated regulation of a large number of alternative splicing events in HeLa cells is consistent with the reported abundant binding of TTP in the intronic RNA regions of mouse macrophages [28]. Considering the central role of NF-κB in immunity, inflammation, and cancer [51, 52], the enrichment of TTP-regulated alternative splicing genes in the I-κB/NF-κB cascade suggests a novel mechanism of TTP in the regulation of the immune response, and that it should be further explored regarding its mechanism as a tumor suppressor. NF-κB is a member of the transcription factor protein family, which includes five subunits: Rel (cRel), p65 (RelA, NF-κB3), RelB, p50 (NF-κB1), and p52 (NF-κB2). In addition, TTP is known to function as part of a negative feedback loop to limit the inflammatory response, including the negative regulation of NF-κB [53]. In the present study, we found that *ZFP36* overexpression was associated with an increase in *RELB* gene expression, which could represent an additional mechanism whereby the NF-κB signaling pathway is regulated.

TTP enriched-regulation of alternatively spliced genes in the TRIF-dependent toll-like receptor, MAPK, TNF, and T cell receptor signaling pathways indicated that TTP-regulated alternative splicing mediated multiple critical biological functions. The TTP-regulated AS genes in these pathways included myocyte enhancer factor 2A (MEF2A), interferon regulatory factor 3 (IRF3), and Toll-like receptor 4 (TLR4) (Fig. 5). In particular, MEF2 has been well-established to contribute to numerous diseases and cancers. Moreover, it has been reported that MEF2A transcripts may include a β exon that increases the capacity of the encoded protein to activate transcription in both striated muscle and neural tissue [54]. Our identification of MEF2A as a TTP-regulated AS gene suggested that MEF2A-dependent transcription regulation might be involved in the immune response.

TLR4 is a member of the TLR family, which is highly expressed in macrophages, dendritic cells, epithelial cells, and B cells. In addition, the alternative splicing of TLR4 has been reported to regulate TLR4 signaling in both mouse and human immune cells [55–57]. IRF3 plays a key role in regulating the innate response against viral infection and IRF3 splicing variants have been reported to affect that ability of IRF3 to trigger the expression of type I interferons and the interferon-stimulated genes in infected cells [58]. Our findings regarding TTP-mediated alternative splicing of TLR4 and IRF3 suggested a more complex network among these immune-response genes.

Because the HeLa cell line is not a cell line exhibiting a normal immune response, its use may limit the biological applications of the findings obtained in this study. However, despite HeLa cells being a cancer cell line, they express genes with diverse biological functions other than tumorigenesis. For example, we showed that TTP and immune response genes were expressed in HeLa cells (74 pathways related to the immune response), at adequate levels when compared with that observed in macrophages in previous studies (for details see Additional file 9). The regulatory mechanisms presented in HeLa cells are likely presented in other cell types, although different cell types may exhibit their own specificity. For example, we have previously reported that a knockdown of PTBP1, a repressor of neuron differentiation, in HeLa cells globally promotes the expression of neuronal genes and neuron differentiation markers [59]. Thus, the regulatory loop established among the PTBP1-microRNA-transcription factors is common in both HeLa and neuronal cell lines [59]. Further studies are required to elucidate the biological relevance of TTP-regulated transcription and the alternative splicing of immune response genes.

## Conclusions

Our study is the first to reveal that TTP extensively regulated the alternative splicing of genes involved in the innate immune response in HeLa cells, which could similarly occur in macrophages and other immune cells. The positive regulation of the gene expression enriched in the type I interferon signaling pathway and viral response has also been demonstrated, which is likely mediated through TTP-mediated binding of DNA targets in the nucleus. Future studies are required to explore the biological function of TTP-mediated alternative splicing, as well as confirm the interaction between TTP and DNA in regulating gene expression. Therefore, these findings revealed an elaborate regulatory TTP-regulatory network connecting transcription, alternative splicing, and mRNA stability, which could play a role in regulating the immune response and tumorigenesis beyond the research in the current study.

## Methods

### *ZFP36* cloning and plasmid construction

Primer pairs used for Hot Fusion were designed by CE Design V1.04 with gene-specific sequences, in conjunction with the portion of the vector pIRES-hrGFP-1a sequences, each with a 17 bp–30 bp overlap.

F-primer: agcccgggcggatccgaattcATGGATCTGACTGCCATCTACG

R-primer: gtcatccttgtagtcctcgagCTCAGAAACAGAGAGGCGATTG

Vector pIRES-hrGFP-1a was digested by EcoRI and XhoI (NEB) at 37 °C for 2 h–3 h. The enzyme-digested vector was also run on a 1.0% agarose gel and purified using a Qiagen column kit. Human RNA was isolated using TRIzol reagent. Purified RNA was reverse transcribed into cDNA using oligo dT primer. Some fragments were synthesized via PCR. A linearized vector digested by EcoRI and XhoI (NEB) and PCR insert (978 bp) were added to PCR microtubes with ClonExpress® II One Step Cloning Kit (Vazyme). Plasmids were introduced into *Escherichia coli* by chemical transformation. The cells were plated onto LB plates containing ampicillin, and the plates were incubated overnight at 37 °C. Colonies were screened by colony PCR (28 cycles) using universal primers (located on the backbone vector). The PCR inert was verified by Sanger sequencing.

### Cell culture and transfection

The human HeLa cell line was purchased from the Institute of Biochemistry and Cell Biology (Chinese Academy of Sciences, Shanghai, China). The cells were seeded into a petri dish (100 mm) at a density of 1 × 10^5^ cells/mL per well, and cultured at 37 °C with 5% CO_2_ in Dulbecco’s Modified Eagle’s Medium (DMEM) containing 10% fetal bovine serum (FBS) (Hyclone), penicillin (100 U/mL), and streptomycin (100 g/mL). The transfection of HeLa cells with a *ZFP36*-overexpressing plasmid was performed using Lipofectamine 2000 (Invitrogen, Carlsbad, CA, USA) according to the manufacturer’s instructions. The transfected cells were harvested after 48 h.

### Assessment of *ZFP36* overexpression

GAPDH (glyceraldehyde-3-phosphate dehydrogenase) was used as a control. cDNA synthesis was performed using standard procedures and real time PCR was performed on the Bio-Rad S1000 with Bestar SYBR Green RT-PCR Master Mix (DBI Bioscience). The concentration of each transcript was then normalized to the level of GAPDH mRNA using the 2- ΔΔCT method [60]. Comparisons were performed with a paired Student’s *t-*test by using GraphPad Prism software (San Diego, CA).

### Western blot analysis

Briefly, to prepare the total cell lysates, normal and *ZFP36*-overexpressing HeLa cells were lysed in RIPA buffer containing 50 mM Tris-HCl (pH 7.4), 150 mM NaCl, 1.0% deoxycholate, 1% Triton X-100, 1 mM EDTA, and 0.1% SDS. The samples were centrifuged (12,000×g for 5 min) and the supernatants were further analyzed on a 10% SDS-PAGE gel and subsequently transferred onto a PVDF membrane (Millipore). TTP was detected using a monoclonal Flag antibody (Sigma-Aldrich) diluted in TBST (1:2000) and Action (Abclonal) was used as the loading control (1:2000).

### Library preparation and sequencing

Total RNA was extracted using TRIzol (Ambion). The RNA was further purified with two phenol-chloroform treatments and RQ1 DNase (Promega) was administered to remove the DNA. The quality and quantity of the purified RNA was assessed by measuring the absorbance at 260 nm/280 nm (A260/A280) using a Smartspec Plus (BioRad). The integrity of the RNA was further verified by 1.5% agarose gel electrophoresis.

For each sample, 1 μg of the total RNA was used for RNA-seq library preparation via VAHTS Stranded mRNA-seq Library Prep Kit (Vazyme). Polyadenylated mRNA was purified and fragmented, and then converted into double stranded cDNA. After the step of end repair and A tailing, the DNAs were ligated to VAHTS RNA Adapters (Vazyme). The purified ligation products corresponding to 200–500 bps were digested with heat-labile UDG, and the single stranded cDNA was amplified, purified, quantified, and stored at − 80 °C before sequencing.

For high-throughput sequencing, the libraries were prepared following the manufacturer’s instructions and applied to the Illumina HiSeq X Ten system for 150 nt paired-end sequencing.

### Real-time qPCR validation of DEGs and AS events

In this study, to elucidate the validity of the RNA-seq data, qPCR was performed for some selected DEGs, and normalized to the reference gene, GAPDH. Information regarding the primers is presented in Table 2. The same RNA samples for RNA-seq were used for qPCR. The PCR conditions consist of denaturing at 95 °C for 10 min, 40 cycles of denaturing at 95 °C for 15 s, followed by annealing and extension at 60 °C for 1 min. PCR amplifications were performed in triplicate for each sample.

Moreover, a qPCR assay was used to analyze the alternative splicing events in HeLa cells. The primers used to detect the pre-mRNA splicing are presented in Table 2. To detect one of the alternative isoforms, one primer was designed in the alternative exon, and an opposing primer was designed in a constitutive exon. To detect the other alternative isoform, a boundary-spanning primer for the sequence encompassing the exon-exon junction with the opposing primer in a constitutive exon was used.

### Bioinformatics analysis and statistical analysis

The adaptors and low quality bases were trimmed from raw sequencing reads by using FASTX-Toolkit (Version 0.0.13). The filtered reads were then aligned to the GRCh38 genome by TopHat2 [61], allowing four mismatches. Uniquely aligned reads were selected to calculate the fragments per kilobase per million mapped reads (FPKM) that represents the expression levels of genes [44]. The software edgeR [45] was utilized to screen for DEGs, based on fold change (FC ≥ 1.5) and false discovery rate (FDR < 0.05). To predict the gene function and calculate the functional category distribution frequency, enriched KEGG pathway and Gene Ontology (GO) terms were identified by using KOBAS 2.0 server [62] and in house tool, respectively. Hypergeometric test and Benjamini-Hochberg FDR controlling procedure were used to define the enriched significance of each pathway (corrected *p*-value < 0.05). The alternative splicing events (ASEs) and regulated alternative splicing events (RASEs) between the samples were defined and quantified using the ABLas pipeline as described previously [49]. After detecting the ASEs in each RNA-seq sample, Fisher’s exact test was used to calculate significant *p*-values, with the alternative reads and model reads of the samples as input data, respectively. The change ratio of alternatively spliced reads and constitutively spliced reads between the compared samples was defined as the RASE ratio. We set *p*-value < 0.05 and RASE ratio > 0.2 as the thresholds for RASE detection.

To explore the mRNA binding profile of TTP, we obtained and analyzed mouse macrophage mRNAs containing TTP binding peaks at the 3′-UTR regions from published data (GSE63466).

## Additional files

Additional file 1: Supporting figures and tables. (DOCX 378 kb)

Additional file 2: Table S2 ZFP36_vs_Ctrl_Sig_DEG. (TXT 135 kb)

Additional file 3: Down_GO_enrichment_P. (XLS 1 kb)

Additional file 4: Up_GO_enrichment_P. (XLS 15 kb)

Additional file 5: Down_KEGG_pathway_iden. (XLS 13 kb)

Additional file 6: Up_KEGG_pathway_iden. (XLS 33 kb)

Additional file 7: ZFP36_vs_Ctrl_NIR_RAS_p0.05. (TXT 147 kb)

Additional file 8: ZFP36_vs_Ctrl_IR_RAS_p0.05_known. (TXT 4 kb)

Additional file 9: Immune_response_gene_FPKM. (XLSX 71 kb)

## Abbreviations

ASEs: Alternative splicing events
Cassette exon: CassetteExon
ES: Exon skipping
FPKM: Fragments per kilo base of exon model per million fragments mapped
MXE: Mutual exclusive exon skipping
PTGR: Post-transcriptional gene regulation
RASEs: Regulated alternative splicing events
RBPs: RNA-binding proteins
TTP: Tristetraprolin
