# Supplementary material for: Tristetraprolin specifically regulates the expression and alternative splicing of immune response genes in HeLa cells
Source: BMC Immunol. 2019 May 2;20:13. doi: 10.1186/s12865-019-0292-1 (PMC6498542; doi:10.1186/s12865-019-0292-1)
Supplement: Supplementary file 1 — Supporting figures and tables. (DOCX 378 kb) [file 12865_2019_292_MOESM1_ESM.docx]

**Supporting Figures and Legends**


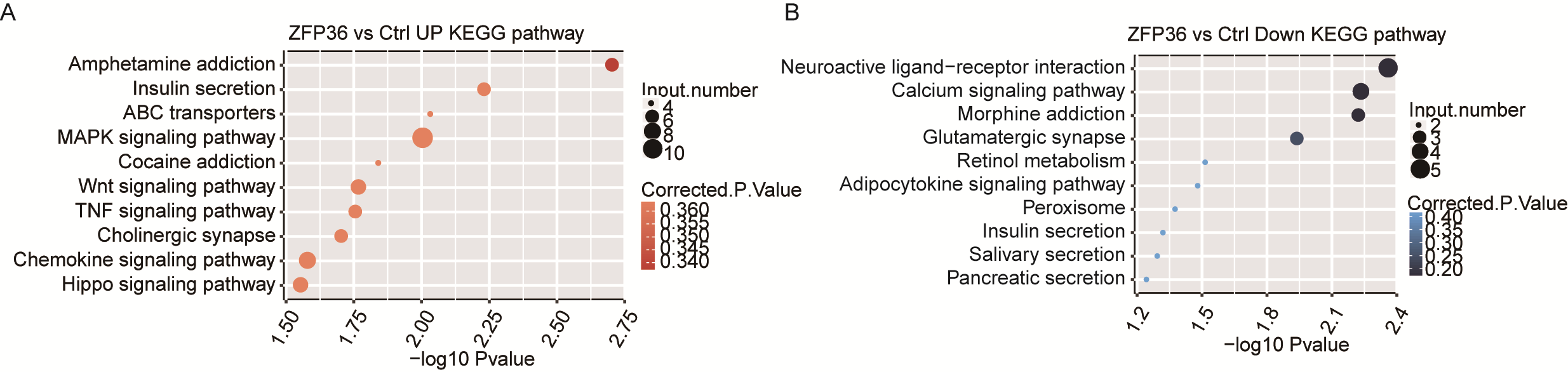


**Figure S1 KEGG pathway analysis of differential expressed genes in HeLa cells after *ZFP36* overexpression.** (A) And (B) Top 10 GO pathway of up-regulated and down-regulated gene sets.


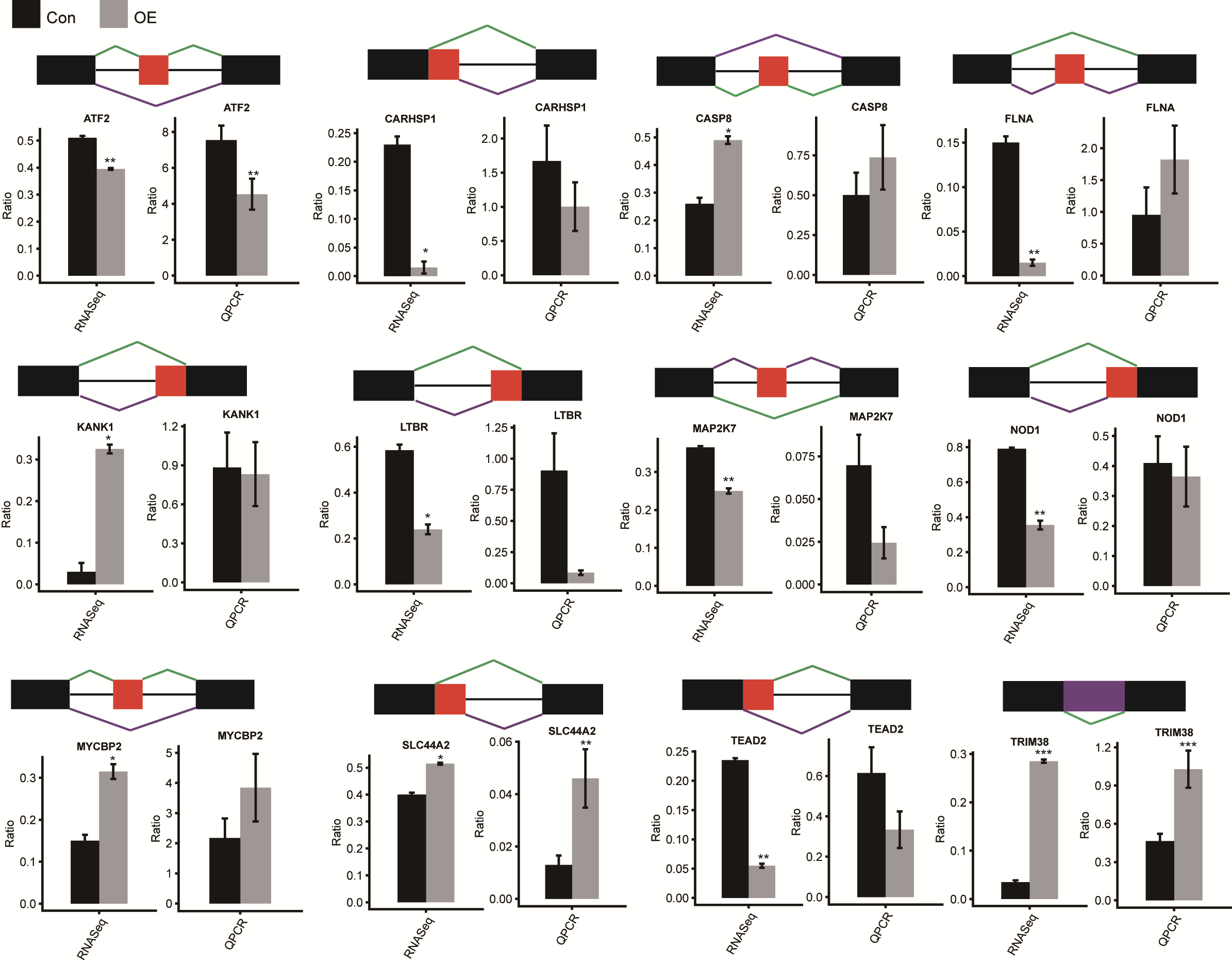


**Figure S2 Validation of ZFP36-affected AS events (continued).** (Top panel) the schematic diagrams depict the structures of alternative splicing events, AS1 (shown in purple) and AS2 (shown in green); exon sequences are denoted by boxes, and intron sequences by the horizontal line. (Bottom panel) RNA-seq quantification and RT-PCR validation of alternative splicing events. The altered ratio of AS events in RNA-seq were calculated using the formula: AS1 junction reads/AS1junction reads +AS2 junction reads; while the altered ratio of AS events in q-PCR were calculated using the formula: AS1 transcripts level/ AS2 transcripts level.

**Supporting Tables and Legend**

**Table S1 the number of gene expressed in four samples**

| Sample | Expressed genes(FPKM>0) | Expressed genes(FPKM>=1) |
| --- | --- | --- |
| Ctrl_1st | 22635 | 12712 |
| Ctrl_2nd | 23692 | 12697 |
| ZFP36_1st | 24417 | 12908 |
| ZFP36_2nd | 23564 | 12776 |
| Total genes | 28837 |  |

**Table S2 Known or novel splicing events detected from ABLas.**

| **Known splicing events** | | | | | | | | | | | | |
| --- | --- | --- | --- | --- | --- | --- | --- | --- | --- | --- | --- | --- |
| Sample | 3pMXE | 5pMXE | A3SS | A3SS&ES | A5SS | A5SS&ES | ES | IntronR | MXE | cassetteExon | Total | Detected junction |
| Ctrl_1st | 342 | 650 | 3222 | 354 | 3666 | 459 | 2481 | 1172 | 311 | 1762 | 14419 | 146811 |
| Ctrl_2nd | 392 | 717 | 3597 | 387 | 4257 | 520 | 2757 | 1214 | 370 | 2022 | 16233 | 150744 |
| ZFP36_1st | 409 | 770 | 3640 | 399 | 4308 | 501 | 2763 | 1233 | 366 | 2040 | 16429 | 153038 |
| ZFP36_2nd | 370 | 721 | 3531 | 397 | 4062 | 519 | 2699 | 1268 | 344 | 1946 | 15857 | 148679 |
| Total | 531 | 1015 | 4837 | 533 | 5668 | 692 | 3503 | 1696 | 540 | 2666 | 21681 | 166260 |
| **Novel splicing events** | | | | | | | | | | | | |
| Sample | 3pMXE | 5pMXE | A3SS | A3SS&ES | A5SS | A5SS&ES | ES | IntronR | MXE | cassetteExon | Total | Detected junction |
| Ctrl_1st | 868 | 2193 | 4608 | 534 | 6108 | 605 | 1920 | * | 331 | 1157 | 18324 | 74418 |
| Ctrl_2nd | 1050 | 2495 | 5219 | 623 | 6759 | 690 | 2153 | * | 375 | 1341 | 20705 | 95700 |
| ZFP36_1st | 985 | 2514 | 5074 | 580 | 6994 | 714 | 2131 | * | 382 | 1374 | 20748 | 98821 |
| ZFP36_2nd | 1043 | 2400 | 5411 | 650 | 7121 | 689 | 2444 | * | 408 | 1288 | 21454 | 84909 |
| Total | 2502 | 5504 | 12801 | 1643 | 16017 | 1759 | 4928 | * | 949 | 3033 | 49136 | 211482 |
| * predicted novel introns are omitted | | |  |  |  |  |  |  |  |  |  |  |
